# Evaluation of Rehabilitation Outcomes in Patients with Chronic Neurological Health Conditions Using a Machine Learning Approach

**DOI:** 10.3390/jfmk9040176

**Published:** 2024-09-26

**Authors:** Gabriele Santilli, Massimiliano Mangone, Francesco Agostini, Marco Paoloni, Andrea Bernetti, Anxhelo Diko, Lucrezia Tognolo, Daniele Coraci, Federico Vigevano, Mario Vetrano, Maria Chiara Vulpiani, Pietro Fiore, Francesca Gimigliano

**Affiliations:** 1 Physical Medicine and Rehabilitation Unit, Sant’Andrea Hospital, Sapienza University of Rome, 00189 Rome, Italy; 2 Department of Anatomical and Histological Sciences, Legal Medicine and Orthopedics, Sapienza University, 00185 Rome, Italy; 3 Department of Biological and Environmental Science and Technologies, University of Salento, 73100 Lecce, Italy; 4 Department of Neuroscience, Section of Rehabilitation, University of Padua, 35122 Padua, Italy; 5 Neurorehabilitation Department, IRCCS San Raffaele, 00163 Rome, Italy; 6 Neurological Sciences and Rehabilitation Medicine Scientific Area, Bambino Gesù Children’s Hospital, 00165 Rome, Italy; 7 Neurorehabilitation Unit, Istituti Clinici Scientifici Maugeri IRCCS, 70124 Bari, Italy; 8 Department of Physical and Mental Health and Preventive Medicine, University of Campania “Luigi Vanvitelli”, 80100 Naples, Italy

**Keywords:** neurological conditions, rehabilitation, Barthel index, artificial neural network, international classification of functioning, mobility, predictive factors

## Abstract

**Background:** Over one billion people worldwide suffer from neurological conditions that cause mobility impairments, often persisting despite rehabilitation. Chronic neurological disease (CND) patients who lack access to continuous rehabilitation face gradual functional decline. The International Classification of Functioning, Disability, and Health (ICF) provides a comprehensive framework for assessing these patients. **Objective:** This study aims to evaluate the outcomes of a non-hospitalized neuromotor rehabilitation project for CND patients in Italy using the Barthel Index (BI) as the primary outcome measure. The rehabilitation was administered through an Individual Rehabilitation Plan (IRP), tailored by a multidisciplinary team and coordinated by a physiatrist. The IRP involved an initial comprehensive assessment, individualized therapy administered five days a week, and continuous adjustments based on patient progress. The secondary objectives include assessing mental status and sensory and communication functions, and identifying predictive factors for BI improvement using an artificial neural network (ANN). **Methods:** A retrospective observational study of 128 CND patients undergoing a rehabilitation program between 2018 and 2023 was conducted. Variables included demographic data, clinical assessments (BI, SPMSQ, and SVaMAsc), and ICF codes. Data were analyzed using descriptive statistics, linear regressions, and ANN to identify predictors of BI improvement. **Results:** Significant improvements in the mean BI score were observed from admission (40.28 ± 29.08) to discharge (42.53 ± 30.02, *p* < 0.001). Patients with severe mobility issues showed the most difficulty in transfers and walking, as indicated by the ICF E codes. Females, especially older women, experienced more cognitive decline, affecting rehabilitation outcomes. ANN achieved 86.4% accuracy in predicting BI improvement, with key factors including ICF mobility codes and the number of past rehabilitation projects. **Conclusions:** The ICF mobility codes are strong predictors of BI improvement in CND patients. More rehabilitation sessions and targeted support, especially for elderly women and patients with lower initial BI scores, can enhance outcomes and reduce complications. Continuous rehabilitation is essential for maintaining progress in CND patients.

## 1. Introduction

Approximately 1 billion individuals worldwide are affected by neurological health conditions, which encompass a broad spectrum of disorders such as stroke, traumatic brain injury, and neurodegenerative diseases [1]. Following the emergence of a neurological disease, individuals frequently encounter difficulties in mobility, which can significantly disrupt their daily activities and diminish their overall quality of life [2]. In the case of sudden-onset neurological health conditions such as stroke, rehabilitation programs targeting motor deficits are typically administered in hospitals or other clinical settings for the first period following their diagnosis [3,4]. While these programs generally offer benefits, it is common for individuals to continue experiencing enduring movement-related impairments even after their completion [3,4,5]. On the contrary, patients with neurodegenerative health conditions, such as Multiple Sclerosis and Parkinson’s disease, are not enrolled in inpatient or outpatient rehabilitation programs until they reach a more advanced stage of the disease, leading to a gradual decline in motor function over time [6,7]. Continual and gradual declines in motor function frequently push individuals with neurological conditions into a cycle of deteriorating health status. These persistent motor deficits interfere with the performance of activities of daily living [8,9,10] and participation in physical activity [10,11]. There are some studies that indicate that a lack of physical activity worsens functioning impairments and contributes to physical deconditioning [12,13,14]. Therefore, the rehabilitation of patients with neurological health conditions should not be perceived as a short-term effort but rather as a lifelong commitment.

In 2001, the World Health Assembly endorsed the “International Classification of Functioning, Disability, and Health” (ICF); this classification system is generally used along with first, second, and third qualifiers [15]. The ICF allows the comparison of data in a biopsychosocial model, considering patients’ body functions and structures, activity and participation (functioning), and the influence of environmental factors [16]. Zhang et al. observed positive correlations between ICF qualifiers in the perspective of body structure, activity participation, and environmental components with clinical assessment tools for stroke, including the Barthel Index [16]. The modified Barthel Index (mBI) was chosen as the primary outcome due to its widespread use and proven reliability in assessing functional independence in activities of daily living (ADLs), particularly in patients with chronic neurological conditions. Its simplicity, validity, and ability to detect clinically meaningful changes make it ideal for evaluating the effectiveness of rehabilitation interventions in this population. Unfortunately, individuals with complex and chronic health conditions encounter numerous obstacles when attempting to engage in rehabilitation, including issues such as therapist availability and financial constraints [17,18,19,20]. In Lazio, as well as in other Italian regions, when a resident citizen needing rehabilitation requires the support of the National Health System, a specific commission has the task to create a so-called Standardized Multidimensional Assessment Schedule (SVaMA) for the patient to develop a project of care tailored to his/her needs (Individual Rehabilitation Plan—IRP) [21].

An artificial neural network is a non-linear computational model composed of input, output, and one or more hidden layers. Each layer’s neurons are interconnected by weighted links, which are continuously adjusted through a training algorithm to reduce errors and enhance prediction accuracy [22]. This approach has been employed to predict the outcomes of physical and rehabilitation therapies [23,24,25,26]. For example, Lin et al. evaluated whether machine learning models could predict the recovery of activities of daily living in acute stroke patients, demonstrating promising results with moderate to high accuracy [27]. Other studies have also assessed machine learning’s potential to predict motor and cognitive improvements in acute and subacute stroke patients, yielding encouraging findings [28,29,30]. However, these studies predominantly focused on inpatient rehabilitation settings for acute or subacute stroke patients. It remains unclear how effectively machine learning methods, particularly neural networks, can predict the response to outpatient rehabilitation interventions, especially in patients with chronic neurological diseases. This gap in knowledge underscores the need for further research to explore the applicability of machine learning in non-hospitalized rehabilitation contexts.

The aims of this study are to describe the characteristics of patients with chronic neurological diseases (PCNDs) who have undergone an IRP without hospitalization to verify whether there is a maintenance or an improvement in outcomes between the start of and discharge from the rehabilitation process, detect any correlations that may be useful in health policies, and identify if there are some descriptive or health status characteristics identifiable at the time of admission that may predict an improvement in the modified Barthel Index (mBI) upon discharge from the rehabilitation project. To do this, an artificial neural network (ANN) was used, which in the rehabilitation medicine field, is becoming relatively competitive with other conventional statistical models [31,32,33,34].

## 2. Materials and Methods

### 2.1. Study Design and Population

We ran an observational study. Our study followed the good clinical practice rules and the Helsinki Declaration, and was approved by La Sapienza University’s Institutional Review Board (RS 0495/2024—Approval Date: 30 May 2024). All patients signed their informed consent, and their data were anonymized before the analysis. Data of patients with chronic neurological health conditions who have undergone an IRP without hospitalization between 2018 and 2023 were retrospectively collected and analyzed. When patients completed more than one IRP over the course of their life with the disease, we considered only data from the last one in chronological order, but still taking into consideration the total number of IRPs performed by each patient. The inclusion criteria were the following: (1) age between 18 and 95 years, (2) IRP in outpatient regimen, (3) presence of a pathology classified in chapter 6 of the International Classification of Disease—9 (ICD 9) “diseases of the nervous system and sense organs (320–389)” for at least 3 months, and of a symptom, sign or disease state belonging to chapter 16 of the ICD 9, or “ill-defined symptoms, signs and disease states (780–799)”, representing the main symptom on which the IRP focuses, and (4) at least one alteration in each of the ICF domains and the assessment of the mBI before and after undergoing the IRP. The exclusion criteria were the following: (1) inpatient care regimes or with overnight stay, and (2) acute phase of the disease.

### 2.2. Intervention

**Figure 1:**
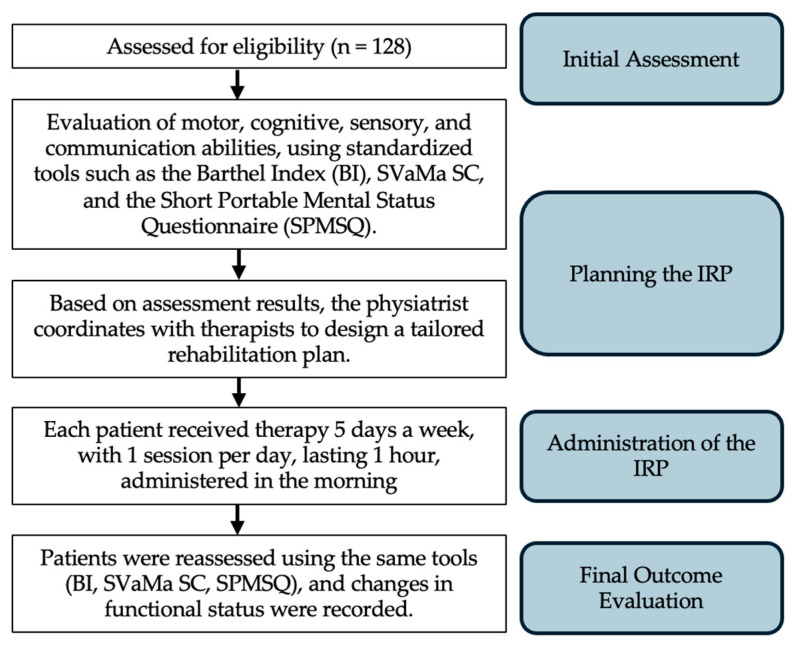
Schematic representation of the IRP administration process from initial assessment to the final outcome evaluation.

The IRP (Individualized Rehabilitation Plan) was designed and tailored by the Rehabilitation Team, coordinated by a physiatrist, and based on the specific needs of each patient. The process involved the following key steps, as shown in Figure 1: (1) Initial Assessment: Upon enrollment in the program, each patient underwent a comprehensive initial assessment, which included the evaluation of motor, cognitive, sensory, and communication abilities, using standardized tools such as the Barthel Index (BI), SVaMa SC, and the short portable mental status questionnaire (SPMSQ). Additionally, the team assessed the patient’s medical history, number of prior IRP programs, and functional limitations according to ICF codes. (2) Planning the IRP: Based on the assessment results, the physiatrist coordinated with therapists (physical therapists, speech therapists, occupational therapists) to design an individualized plan. (3) Administration of the IRP: each patient received therapy 5 days a week, with 1 session per day, lasting 1 h, and administered in the morning [35]. Therapists continually monitored patient progress throughout the intervention period, adjusting the therapy content and intensity as needed based on the patient’s response and improvement in function. (4) Final Evaluation: upon completion of the IRP, patients were reassessed using the same tools (BI, SVaMa SC, SPMSQ), and changes in functional status were recorded. The final Barthel Index score at discharge was used as the primary outcome measure to evaluate the effectiveness of the rehabilitation intervention.

### 2.3. Assessment and Outcome Measures

The SVaMA is a multidimensional evaluation scale; it includes the assessment of physical functions using the modified Barthel Index (mBI) [36], of sensory and communication functions (including language understanding (Lc) and production (Lp), hearing (U), and sight (S); this part is named SVaMA SC) [37,38], and of other cognitive functions using the short portable mental status questionnaire (SPMSQ). Therefore, we have 3 scores: the mBI, the SVaMA SC score, and the SPMSQ score.

The Barthel Index was first published in 1965 and consists of 10 items [39]. It was further improved by standardizing the rating criteria and scale into a five-point Likert scale format [40]. The mBI has increased the sensitivity of the instrument both at the item and scale levels, and it has yielded a greater content reliability and internal consistency [36,41,42,43,44]. The mBI consists of 10 daily activity items and can be analyzed using a total score ranging from 0 to 100. Each item is scored on a 5-level scale, from 1 (completely dependent) to 5 (completely independent) [40].

The SVaMA scale assesses multiple areas of communication and sensory abilities. The SVaMA SC scores range from 0 to 3 based on normality or complete impairment. In terms of “language understanding”, it categorizes individuals as either having normal understanding, being able to understand only simple sentences, or not understanding at all. For “language production”, individuals are classified as having unimpaired speech, a language disorder, or an inability to speak. “Hearing” is assessed on a spectrum from unimpaired hearing to having a serious deficit, or being deaf. “Sight” is evaluated as unimpaired, having a serious deficit, or being blind. And an item corresponding to the total sum of the scores from the various items [45].

The SPMSQ is a widely recognized cognitive screening tool that assess various aspects of cognitive function (orientation to time and place, memory, current event information, calculation). It consists of 10 items aiming to assess cognitive impairment. Scores range from 0 to 10, and it is possible to identify 3 groups: no/mild cognitive impairment < 3 mistakes, moderate 3 to 4 mistakes, and severe ≥ 5 mistakes [46]. The validity and reliability of SPMSQ as an effective cognitive screening instrument was established in several studies [47,48,49,50,51].

The ICF has two parts, each containing two components. The first part deals with functioning and disability and includes the body function (b) and body structure (s) components, as well as the activities and participation (d) component. The second part covers contextual factors and includes the environmental factors (e) component and the personal factor component. Each component includes several categories, which are the units of the ICF classification [5,15,52,53,54].

### 2.4. Statistical Analysis

For the sample size calculation, the software g*power vers. 3.1.9.7 was used. We based our calculations on previous results obtained by Masanori Okamoto et al. [55]. For a two-tailed test with an α level of 0.05, 95% confidence interval (CI), and a statistical analysis of the desired power of 90% (error β = 10%), a minimum sample size of 116 people was obtained. All data points were included in the descriptive statistical calculations. Data were analyzed using SPSS Statistics version 27 (IBM SPSS, IBM Corp., Armonk, NY, USA) [56]. The mean and SD of any interval data (e.g., years from pathology, admission modified Barthel Index) and the frequency of any categorical data (e.g., gender or categories of International Classification of Functioning) were calculated, as well as the bivariate correlations. Descriptive statistics for various physiatrist-assessed outcome measures (PAOMs) were calculated. The mean and standard deviation (SD) of the interval data, the frequency of categorical data, and the bivariate correlations were calculated. An independent samples *t*-test was conducted to compare the initial levels of cognitive impairment (SPMSQ) between males and females. An independent samples *t*-test was conducted to compare the delta (Δ) mBI between males and females. The ΔmBI at discharge was coded as a binary variable, with 1 indicating improvement and 0 indicating maintenance or worsening [57,58]. The formula used was ΔmBI = Discharge mBI—an admission mBI > 0 was defined as improved and ≤0 was defined as unimproved [59]. A General Linear Model (GLM) with repeated measures was conducted, employing “time” as the within-subjects factor, which consisted of two levels corresponding to the “admission SPMSQ” and “discharge SPMSQ”. Additionally, we included the “binary value of improvement mBI” as a between-subjects factor. A difference with *p* < 0.05 was considered statistically significant.

### 2.5. Artificial Neural Network

The ANN model was developed using SPSS 27.0 statistical software by SPSS Inc. in Chicago, Illinois [56]. The ANN analysis aimed to identify influential variables and model their impact on the mBI at discharge of patients with chronic neurological health conditions who have undergone an IRP without hospitalization. The chosen model was the multilayer perceptron (MLP), which comprises three layers: input layer, hidden layer, and output layer [23]. To ensure the robustness and generalizability of the model, we implemented a 5-fold cross-validation approach [60,61,62]. The MLP ANN utilized predictive factors that could be collected before the start of the IRP within the input layer with the independent variable, including demographic factors (age; gender), clinical variables (Macroarea of Pathology; ICF CODES S, B, D1 AND D2, E1, and E2), treatment factors (planned sessions of neuromotor rehabilitation; number of rehabilitation projects per patient; planned days of absence; years from pathology), physiatrist-assessed outcome measures (PAOMs) (admission SVaMA SC total; threshold ≤ 30 points mBI admission [63,64,65]; threshold ≤ 45 points mBI admission [66,67,68]), admission short portable mental status questionnaire (SPMSQ), and the output layer (improvement in mBI or not), to learn the intricate relationship between the inputs and output (Table 1). The modified Barthel Index was chosen as the outcome variable of interest because it is significantly associated with independence in personal ADLs [69], previous research shows that mBI is impacted by chronic neurological diseases (CNDs), and the mBI is a primary goal of the rehabilitation program [70]. The output layer featured two neurons with a target error of 0.0001, a learning rate of 0.001, and a maximum training period of 1000 iterations, and training concluded upon reaching the minimum error value. In our study, patients were randomly assigned to two groups: 80.4% for the total training sample and 19.6% for the validation sample (testing group) [71]. These subsets were employed in developing the ANN models. After training the MLP ANN, it was employed to predict outcomes using the test subset.

## 3. Results

### 3.1. Demographics

Most patients had completed more than one IRP over the course of their life with the disease. In total, 128 patients completed 534 rehabilitation projects over the course of their life with the disease. In this study, we considered the last one in chronological order, but considering in the results the number of IRPs performed by each of the patients with chronic neurological diseases.

After applying the inclusion and exclusion criteria, 128 patients with 534 rehabilitation projects for their chronic neurological disease were finally included in the study. Concerning gender, the majority were female (n = 71, 55.5%), and the males were fewer (n = 57, 44.5%). Also, if we considered here just one IRP for each patient, there are some patients that completed more than one IRP; in total, the 128 patients here examined completed 534 projects, with a mean of 4172 and ±3. The mean age was 48.9 (±10.5) years. The patients, before starting the IRP, were assessed with items from the ICF, and in relation to demographic data, we inserted here the qualifiers regarding environmental factors represented by “E codes”. Two codes were collected for category E for a total of 256 values. We analyzed data pertaining to the category “ICF E—Environmental Factors”. One significant facilitator identified is the use of general products and technology for personal daily living (e115.+3). These tools are reported to have a severe level of facilitation, indicating their crucial role in enabling individuals to carry out daily activities independently. This facilitator is observed with a frequency of 45%. Additionally, facilitation at a moderate level (e115.+2) suggests that while some assistance may be required, individuals still benefit from the use of these products and technologies. This level of facilitation is observed with a frequency of 30%. Moreover, family support emerges as a fundamental facilitator across different levels of severity. Immediate family members (e310) provide significant facilitation (e310.+3), indicating a crucial support network that aids individuals in various aspects of their lives. This support is reported with a frequency of 60%. Additionally, support from family members at a moderate level (e310.+2) is observed with a frequency of 40%, while support at a mild level (e310.+1) is observed with a frequency of 15%. Furthermore, assistance from caregivers and healthcare providers plays a crucial role in facilitating individuals’ functioning. Both family members and other caregivers (e340.+3) provide significant support, marked as severe facilitation, indicating their essential role in assisting individuals with disabilities. This level of facilitation is observed with a frequency of 55%. Additionally, healthcare providers (e360.+3) outside the immediate family are also reported as severe facilitators, demonstrating the broader support network involved in facilitating individuals’ functioning. This level of facilitation is observed with a frequency of 25%.

### 3.2. Physiatrist-Assessed Outcome Measures

Physiatrist-assessed outcome measures of the study population are shown in Table 2. The mean modified Barthel Index (mBI) score at discharge (42.53 ± 30.02) was significantly (*p* < 0.001) better than at baseline (40.28 ± 29.08). At discharge, 62 patients showed an improvement in the mBI, 50 maintained their scores, and 16 had a worsening.

In the SVaMA Sensory and Communication total, including production of language (Lp), hearing (U), and sight (S), there were no statistically significant differences. However, for understanding of language (Lc), there was a significant (*p* < 0.05) improvement at discharge (2.52 ± 0.9) compared to baseline (2.56 ± 0.7).

The mean short portable mental status questionnaire (SPMSQ) score at discharge (3.8 ± 4.4) was significantly (*p* < 0.05) better than at baseline (3.5 ± 4.5). Dividing the admission score into three levels, it was found that women had a higher SPMSQ (1.48 ± 0.5) compared to men (1.33 ± 0.4) (*p* < 0.05), corresponding to greater cognitive deterioration.

Data pertaining to the category “ICF B—Body Functions” were analyzed. The impairment recorded that “Severe impairment of muscle strength function” (ICF code: b730.3) was prevalent, noted in a substantial proportion of cases (69.72%). Furthermore, “Complete impairment of muscle strength function” (ICF code: b730.4) was also notable, documented in 20.27% of cases. Additionally, impairments related to “Severe impairment of muscle tone function” (ICF code: b735.3) and “Severe impairment of joint mobility function” (ICF code: b710.3) were identified in 2.03% and 3.39% of cases, respectively.

Data pertaining to the category “ICF S—Structure” were analyzed. The impairment “Complete impairment of structures of the nervous system” (ICF code: s110.40) was the most frequently encountered (64.14%). Furthermore “Complete impairment of spinal cord and related structures” (ICF code: s120.40) was observed in 20.27% of cases. “Severe impairment of trunk structures” (ICF code: s760.30) and “Severe impairment of lower limb structures” (ICF code: s750.30) were identified in 3.39% and 2.03% of cases, respectively.

Data pertaining to the category “ICF D—Mobility” were analyzed, focusing on the most frequently encountered activities and their associated levels of difficulty. The activity “Walking” (ICF code: d450) was identified as prevalent in 32.73% of cases, indicating a significant level of difficulty, often requiring complete assistance. Similarly, “Transferring oneself” (ICF code: d420) was observed in 25.45% of cases, with a moderate level of difficulty ranging from partial to complete assistance. Lastly, “Speaking” (ICF code: d330) was present in 9.09% of cases, typically associated with severe or complete difficulty.

### 3.3. Clinical and Rehabilitative Information

**Figure 2:**
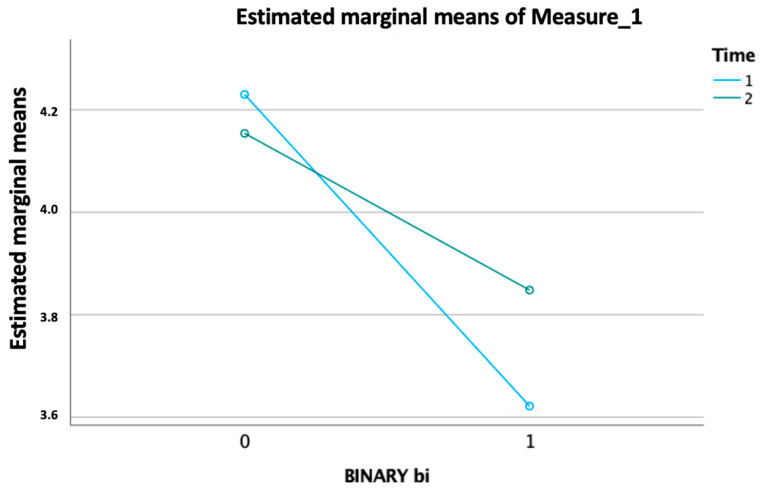
Significant effect of improvement in the modified Barthel Index over time, with intersecting trends for ‘admission SPMSQ’ and ‘discharge SPMSQ’ (*p* < 0.05).

Clinical and rehabilitative information of the study population are shown in Table 3. Analyzing the patients, we found that macroareas of the most frequently detected disease were non-specified quadriplegia in 30.4% of the patients; paraplegia, diplegia, and monoplegia in 29.6% of the patients; pathology of the basal ganglia in 22.6% of the patients; pathology of the myelin in 8.6% of the patients; and senile degenerative brain in 2.3% of the patients. Patients had an average duration of pathology of 19.172 years ± 16.1. During the IRP, patients underwent an average of 43.73 ± 17.6 planned neuromotor rehabilitation sessions. During the rehabilitation project, the patients had an average of days of absence of 3.7 ± 4.4. Regarding the average SPMSQ at admission in patients: the 28 males who improved in mBI had an average admission score of 2.8; the 29 males who did not improve in mBI had a mean admission score of 3.4; the 34 females who improved in mBI had an average admission score of 3.5; the 37 females who did not improve their BI had a mean admission score of 4.7. Most patients had a statistically significant improvement (*p* value < 0.05) in their mBI; specifically, 48% of patients improved and 39% maintained the progress made in the past. The GLM analysis revealed a significant main effect for the “binary value of improvement modified Barthel Index” per “time” as the within-subjects factor, which consisted of two levels corresponding to the “admission SPMSQ” and “discharge SPMSQ” (*p* < 0.05) (Figure 2).

**Figure 3:**
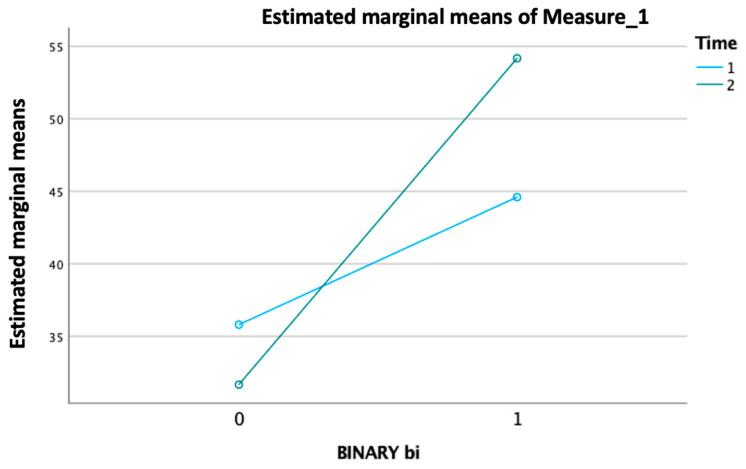
Significant effect of improvement in the modified Barthel Index over time, with distinct trends for ‘admission’ and ‘discharge’ levels (*p* < 0.001).

Another GLM analysis revealed a significant main effect for the “binary value of improvement modified Barthel Index” per “time” as the within-subjects factor, which consisted of two levels corresponding to the “admission modified Barthel Index” and “discharge modified Barthel Index “ (*p* < 0.001) (Figure 3).

Correlation analysis showed several significative interactions that were confirmed by linear regression between the following: 

(1) the age and the admission SVaMA SC Tot at admission and discharge (r = 0.377, *p* < 0.001 and r = 0.347, *p* < 0.001), indicating that as the age increases, the SVaMA SC Tot at admission and discharge increases; to further confirm this relationship, a linear regression between them was performed. The regression results showed that age is a significant predictor of SVaMA SC Tot at admission (β = 0.05, t = 4.5, *p* < 0.001) and SVaMA SC Tot at discharge (β =0.05, t = 4.1, *p* < 0.001), explaining a significant proportion of the variance in both SVaMA SC Tot at admission and discharge (R^2^ = 0.1). This result suggests that older patients tend to have higher SVaMA SC Tot scores at admission and discharge.

(2) The Barthel Index at discharge and the number of days absent from the IRP (r = −0.44, *p* < 0.001), indicating that as the Barthel Index at discharge increases, the number of days absent decreases; to further confirm this relationship, a linear regression between them was performed. The regression results showed that the Barthel Index at discharge is a significant predictor of the number of days absent (β = −0.065, t = −5.4, *p* < 0.001), explaining a significant proportion of the variance in the number of days absent (R^2^ = 0.2). This result suggests that patients with a higher Barthel Index at discharge tend to have fewer days absent from the IRP.

(3) The age and the number of days absent from the IRP (r = −0.287, *p* < 0.001), indicating that as the age increases, the number of days absent decreases; to further confirm this relationship, a linear regression between them was performed. The regression results showed that age is a significant predictor of the number of days absent (β = −0.072, t = −3.3, *p* < 0.001), explaining a significant proportion of the variance in the number of days absent (R^2^ = 0.08). This result suggests that older patients tend to have fewer days absent from their IRP.

(4) The age and the planned sessions of neuromotor rehabilitation (r = −0.271, *p* < 0.002), indicating that as the age increases, the planned sessions of neuromotor rehabilitation decrease; to further confirm this relationship, a linear regression between them was performed. The regression results showed that age is a significant predictor of the sessions of neuromotor rehabilitation (β = −0.27, t = −3.1, *p* < 0.002), explaining a significant proportion of the variance in the sessions of neuromotor rehabilitation (R^2^ = 0.75). This result suggests that older patients tend to perform fewer sessions of neuromotor rehabilitation.

(5) The Barthel Index at discharge and the number of planned sessions of neuromotor rehabilitation (r = −0.226, *p* < 0.01), indicating that as the Barthel Index at discharge increases, the sessions of neuromotor rehabilitation decrease; to further confirm this relationship, a linear regression between them was performed. The regression results showed that the Barthel Index at discharge is a significant predictor of the sessions of neuromotor rehabilitation (β = −0.132, t = 2.6, *p* < 0.01), explaining a significant proportion of the variance in the number of sessions of neuromotor rehabilitation (R^2^ = 0.5). This result suggests that patients with a lower Barthel Index at discharge tend to perform more sessions of neuromotor rehabilitation.

### 3.4. Predicting an Improvement in Barthel Index at Discharge from the Rehabilitation Project with an ANN

Performance and influential factors of the ANN model for mBI prediction are shown in Table 4. The use of machine learning can help in predicting mBI and improving the rehabilitation process accurately [23]. An ANN (artificial neural network) model was developed using SPSS 27.0, a statistical software by SPSS Inc. in Chicago, Illinois [56]. The ANN analysis aimed to identify influential variables and model their impact on mBI over discharge from the rehabilitation project. The chosen model was the multilayer perceptron (MLP), which comprises three layers: the input layer, hidden layer, and output layer. The MLP ANN utilized predictive factors within the input layer as factors—gender; macroarea of pathology; ICF Code B, S, D, E; total sum of admission SVaMA SC; and two thresholds of the mBI in admission at 30 [63,64,65] and 45 points [66,67,68] of the score—and as covariates—planned sessions of neurorehabilitation, the score of SPMSQ at admission, years from pathology, age, and the sum of past rehabilitation project performed. As the output layer, the binary values of mBI improvement allow the observation of the intricate relationship between inputs and output. The output layer features a single neuron. In our study, patients were randomly assigned to two groups: from the total sample, 80.4% were assigned to the training group, and the remaining 19.6% constituted the testing group. These subsets were employed in developing the ANN models. After training the MLP ANN, it was employed to predict outcomes using the test subset. The multilayer perceptron network developed a percentage of incorrect predictions of 13.6% in the test and therefore an overall accuracy of 86.4%. The area under the receiver operating characteristic (ROC) curve was of 0.729, and this is a significant result because the area under the curve (AUC) has a value between 0 and 1, but is meaningful as a diagnostic test only when it is >0.5 [72]. The percentage weight of each factor is 100% for the ICF D CODE, 94.7% for the ICF B CODE, 94.5% for the number of rehabilitation projects performed, 91% for the ICF S CODE, 91% for the macroareas of pathology, 83.8% for the planned sessions of neuro-motor rehabilitation, 77.8% for the E CODE, 74.7% for the years from pathology, 48.2% for the total sum of SVaMA Sc admission, 45.9% for the admission SPMSQ, 45.5% for the age of the patients, 25.9% for the thresholds of mBI admission >30, 17.2% for the threshold of mBI admission > 45, and 15.9% for gender. With SPSS 27.0 statistical software by SPSS Inc. in Chicago, Illinois, through descriptive statistics with crosstabs, we assessed the values in percentage of the positive predictive value (73.7%), of sensibility (71.2%), of negative predictive value (69.1%) and of specificity (71.7%), as well as the overall accuracy of 86.4%.

## 4. Discussion

The recent advancements in AI hold the potential to serve as a powerful instrument for conducting a more profound examination of predictive elements through the utilization of machine learning techniques [24,25,73,74]. The primary distinction from conventional statistical methods lies in how ANN allocates a weight to each of the factors under investigation. As a result, AI has the potential to offer a more intricate yet also a more comprehensive insight into comprehending the factors affecting neurorehabilitation outcomes within a diverse population, like patients with chronic neurological diseases (PCNDs) [75]. Our ANN developed an overall accuracy of 86.4%, in line with other machine learning approaches (including random forest, gradient boosting, support vector machines, decision trees, and k-nearest neighbors), as reported in a recent review about their use in stroke rehabilitation [74].

Our analysis, using artificial neural networks, identified the D code of the ICF, related to the chapter ‘Activities and Participation’, as having a high predictive value for improvement in the Barthel Index. This finding aligns with the existing literature that mobility deficits are associated with decreased quality of life [76], disability in daily activities [77], risk of falls [78], hospitalization [79], and mortality [80]. The high predictive value of mobility-related qualifiers in our study suggests that the difficulty to mobilize significantly predicts improvements in daily life activities. These results emphasize the need to focus on enhancing mobility in rehabilitation programs for patients with chronic neurological disorders to improve their overall functional outcomes.

As described by Völter et al., the elderly often suffer a marked decline in one or more sensory systems [81]. In fact, we detected a significative bivariate correlation between age and total SVaMA Sc score, as they increase at the same time, which means that with the elderly, sensory and linguistic problems increase.

The substantial rise in hospital costs over recent years is associated with the rapid increase in the older age population [82]. Given the detection of a significant improvement in the mBI, we can deduce that it is useful for the National Health System to finance rehabilitation programs for patients with chronic neurological diseases (PCNDs), with the aim of allowing access to everyone who needs more, even those who cannot financially support these paths. This will probably lead to a decrease in public health spending linked to the complications of chronic neurological diseases (CNDs). In other areas of rehabilitation, a high number of sessions with physiotherapists is associated with better outcomes compared to participation in a lower number of sessions [83,84]. This justifies the high weight as a percentage of the planned number of neuromotor rehabilitation sessions detected in our study, suggesting that a high number of rehabilitation sessions, albeit more demanding, leads to better outcomes, especially with regard to daily living activities.

Attention must also be paid to the moderate percentage weight of the ICF E code in predicting an improvement in the mBI and, therefore, the daily life of patients with chronic neurological diseases. As proposed by Kemp et al. in “Improving wellbeing in patients with chronic conditions: theory, evidence, and opportunities” [85], we investigated and found that these qualifiers, corresponding to environmental factors relating mainly to products and technology, as well as support and relationships, are implicated in improving the BI, and therefore independence in personal activities of daily living.

As reported, nutritional status is associated with poor rehabilitation outcomes [86], and in our study, the qualifiers about food were reported 10 times as a barrier for these patients, and the average improvements in the BI at discharge were just of 4 to 6 points for these patients, concordant with the study of Wakabayashi et al. [86].

The SPMSQ is used as a test for dementia [84]; it has been found that dementia tends to be more frequent in elderly women [87], and in our study, we confirm this result. The included women had a significative higher average SPMSQ level at admission than men, corresponding to greater cognitive deterioration (1.48 DS 0.5 vs. 1.33 DS 0.4) (*p* < 0.05). Furthermore, the 28 males who improved in their mBI had a superior average improvement in mBI of 12.25 DS 7.9 points, while the 34 females who improved their mBI had an average improvement in mBI of 7.6 DS 4.6 points (*p* < 0.05). Considering that previous research highlighted that women develop greater cognitive impairments and problems during neurological pathologies, particular in stroke [88], our results align with this research, and to confirm it, we made a correlation test between the SPMSQ in males and women with respect to the Delta Barthel Index. While in men, the correlation did not appear to exist, in women, there was a weak correlation (R = −0.3), which was statistically significant (*p*= 0.03). This means that as the SPMSQ increases in women, there is a decrease in the Delta Barthel Index, and therefore a decrease in the improvement in the effectiveness of the rehabilitation intervention. This finding allows us to hypothesize that a greater cognitive impairment also causes a lesser improvement in the mBI of elderly women.

The findings from the GLM analysis highlight the significant association between improvement in Barthel Index scores and changes in SPMSQ scores over time. Our results suggest that individuals with initially higher SPMSQ scores, indicative of better cognitive function, were more likely to experience improvement in their Barthel Index scores (*p* < 0.05). This observation underscores the potential influence of cognitive status on functional outcomes in this population. Our findings can be supported by various studies that highlight the link between cognitive function and rehabilitation outcomes. For example, a study found that improvements in cognitive function, as measured by tools like the SPMSQ, were significantly correlated with functional independence, as measured by the Barthel Index [89]. Another research demonstrated that cognitive impairments, such as unilateral neglect and anosognosia, negatively affected outcomes in rehabilitation programs [90].

The GLM analysis further demonstrated a significant main effect for the ‘binary value of Improvement Barthel Index’ across ‘time’ as the within-subjects factor, which included levels corresponding to the ‘admission Barthel Index’ and ‘discharge Barthel Index’ (*p* < 0.001). These results underscore the importance of initial functional status as a predictor of rehabilitation outcomes. Patients with higher initial Barthel Index scores were more likely to experience significant improvements, suggesting that functional status at admission in chronic neurological pathology plays a crucial role in the trajectory of rehabilitation success. Our findings regarding the significant association between better initial Barthel Index (BI) scores and better rehabilitation outcomes are supported by several articles [90,91,92].

Neurological conditions affect various age groups differently, and older patients may present distinct clinical and functional challenges compared to younger ones. Therefore, by including a broader age range, we aimed to assess the variability in rehabilitation outcomes across the lifespan, which could provide insights for more personalized and age-specific rehabilitation interventions in future studies [93,94,95].

The results of a correlation analysis confirmed by linear regressions suggest that older patients tend to have higher impairment in sensory and communication function and body structures at admission and discharge. Older patients tend to attend fewer days of neuromotor rehabilitation, but they also tend to have fewer days of absence from the rehabilitation program. The elderly are probably more adherent to therapies as they age but can perform fewer of them because family caregivers are often extremely fatigued. In particular, those who have no respite risk their well-being and their ability to provide care [96], causing a decrease in adherence to therapy, as seen in our findings. Therefore, it would be desirable to increase the number of physiotherapists, dedicated nurses, and logistical assistants, such as drivers and transporters, to allow patients with chronically severe neurological disabilities to attend rehabilitation facilities. Patients with a higher Barthel Index at discharge tend to have fewer days of absence from the rehabilitation program. Patients with a lower Barthel Index at discharge tend to perform a higher number of neuromotor rehabilitation sessions, probably due to more severe conditions.

## 5. Limitations

The primary limitation of this study is its relatively small sample size, which may limit the generalizability of the findings. The methodological procedures employed in this retrospective observational study included extracting patient data from a pre-existing dataset. While this approach allows for the analysis of a broad range of conditions, it also presents several limitations: the use of a pre-existing dataset means that the quality and completeness of the data were dependent on the original data collection processes. Missing or incomplete data may have impacted the accuracy of the analysis. Applying inclusion and exclusion criteria to the dataset may introduce selection bias. The criteria used to standardize the population might not fully account for the variability within the broader population of neurological disorders, potentially limiting the representativeness of the sample. As a retrospective study, a randomization of participants and intervention assignments was not possible, which may limit the ability to draw causal inferences about the effectiveness of the interventions. The variability in therapies administered to patients during the IRP (e.g., the amount of time spent on physical therapy versus occupational therapy) was not fully accounted for in this study. The artificial neural network (ANN) generalizes across the dataset to identify broader patterns, potentially overlooking individual differences in therapy intensity and type. This generalization represents an inherent bias in the study, as rehabilitation is highly personalized, and outcomes may vary based on the specific intervention administered. Future research should include larger observational studies with additional variables and randomized controlled trials to more comprehensively evaluate the effectiveness of rehabilitation strategies for chronic neurological patients. These studies should also consider analyzing the specific impact of the therapy type and duration to ensure a more accurate reflection of rehabilitation outcomes in diverse patient populations, and should also focus on more specific subgroups within this population to better understand how age and specific neurological diagnoses affect rehabilitation outcomes.

## 6. Conclusions

Artificial neural networks (ANNs) achieved an accuracy comparable to other machine learning methods, providing deeper insights into factors influencing neurorehabilitation outcomes in patients with chronic neurological diseases (PCNDs). AI can offer a more complex understanding of factors affecting rehabilitation success by assigning different weights to each factor, thus enhancing outcome prediction. The ICF “Activities and Participation” (D codes) showed a high predictive value for improvement in the Barthel Index (BI), emphasizing the importance of addressing mobility deficits in rehabilitation to improve daily activities and quality of life. A significant correlation between age and sensory/linguistic problems was found, with older individuals showing increased impairment in these functions, confirming the literature findings. Continued funding for rehabilitation programs for PCNDs is justified, as improvements in the BI were observed, potentially reducing public healthcare costs by preventing complications associated with chronic neurological diseases. A higher number of rehabilitation sessions was associated with better outcomes, particularly regarding activities of daily living, underscoring the need for intensive rehabilitation in PCNDs. The moderate predictive weight of ICF “Environmental Factors” (E codes), related to technology, products, and support systems, indicates their role in improving independence in daily activities for PCNDs. Nutritional status was identified as a barrier to rehabilitation progress, with poor nutritional health linked to smaller improvements in the BI. Elderly women showed greater cognitive deterioration (higher SPMSQ scores) and had smaller improvements in BI than men, suggesting a correlation between cognitive impairment and poorer rehabilitation outcomes in women. Patients with better initial cognitive function (lower SPMSQ scores) demonstrated better improvements in the BI, reinforcing the impact of cognitive status on functional recovery. Higher initial BI scores predicted better rehabilitation outcomes, highlighting the importance of initial functional status for the success of rehabilitation programs. Older patients, despite performing fewer rehabilitation sessions, showed higher adherence, likely due to the involvement of family caregivers, who may require additional support to prevent burnout and maintain patient adherence. Increasing the number of physiotherapists, nurses, and logistical support personnel would improve access to rehabilitation services, especially for those with severe neurological disabilities, to enhance attendance and outcomes.

## Figures and Tables

**Table 1:** Inputs and output of the ANN model.

Layer	Variables
**Input Layer**	
Demographic Factors	- Age - Gender
Clinical Variables	- Macroarea of Pathology - ICF CODES S, B, D1 AND D2, E1 and E2
Treatment Factors	- Planned sessions of neuromotor rehabilitation - Number of rehabilitation projects per patient - Planned days of absence - Years from pathology
Physiatrist-Reported Outcome Measures (PROMs)	- Admission SVaMA SC Total - Threshold ≤ 30 points mBI admission - Threshold ≤ 45 points mBI admission - Admission short portable mental status questionnaire (SPMSQ)
**Output Layer**	- Improvement in mBI or not

**Table 2:** Patient characteristics and ICF category analysis.

Category	Details
**Modified Barthel Index (mBI)**	**Baseline:** 40.28 (±29.08) **Discharge:** 42.53 (±30.02) **Improvement:** *p* < 0.001
**mBI Outcomes**	**Improvement:** 62 patients **No change:** 50 patients **Worsening:** 16 patients
**SVaMA Sensory and Communication Total**	No significant differences in Lp, U, and S
**Understanding of Language (Lc)**	**Baseline:** 2.56 (±0.7) **Discharge:** 2.52 (±0.9) **Improvement:** *p* < 0.05
**SPMSQ Score**	**Baseline:** 3.5 (±4.5) **Discharge:** 3.8 (±4.4) **Improvement:** *p* < 0.05
**SPMSQ Score admission levels Gender differences**	**Female:** 1.48 ± 0.5 **Male:** 1.33 ± 0.4 (*p* < 0.05)
**ICF B** **—** **Body Functions**	Severe muscle strength impairment (b730.3) 69.72% Complete muscle strength impairment (b730.4) 20.27%
**ICF S** **—** **Structure**	Complete impairment of structures of the nervous system (s110.40) 64.14% Complete impairment of spinal cord and related structures (s120.40) 20.27%
**ICF D** **—** **Mobility**	Difficulty in walking (d450) 32.73% Difficulty in transferring oneself (d420) 25.45% Difficulty in speaking (d330) 9.09%

**Table 3:** Analysis of patient characteristics and rehabilitation outcomes.

**Disease Macroareas**	Non-Specified Quadriplegia: 30.4% Paraplegia, Diplegia, Monoplegia: 29.6% Basal Ganglia Pathology: 22.6% Myelin Pathology: 8.6% Senile Degenerative Brain: 2.3%
**Duration of Pathology**	Mean: 19.172 years (±16.1)
**Rehabilitation Sessions**	Planned Neuromotor sessions: Mean 43.73 (±17.6)
**Days of Absence**	Mean: 3.7 days (±4.4)
**SPMSQ Scores (Males)**	Improved (n = 28): Mean admission score 2.8 Not improved (n = 29): Mean admission score 3.4
**SPMSQ Scores (Females)**	Improved (n = 34): Mean admission score 3.5 Not improved (n = 37): Mean admission score 4.7
**mBI Improvement**	Improved: 48% Maintained: 39% Statistically significant improvement (*p* value < 0.05)
**GLM Analysis**	Significant effect for binary value of improvement mBI by time (admission and discharge SPMSQ) (*p* < 0.05) Significant effect for binary value of improvement mBI by time (admission and discharge mBI) (*p* < 0.001)

**Table 4:** Performance and influential factors of the ANN model for mBI prediction.

Model Type	Multilayer Perceptron (MLP)
**Training Group**	80.4% of total sample
**Testing Group**	19.6% of total sample
**Model Accuracy**	86.4%
**Incorrect Predictions**	13.6%
**Area Under ROC Curve (AUC)**	0.729
**Influential Factor Weights**	ICF D Code: 100% ICF B Code: 94.7% Rehabilitation Projects Performed: 94.5% ICF S Code: 91% Macroareas of Pathology: 91% Planned Sessions of Neurorehabilitation: 83.8% ICF E Code: 77.8% Years from Pathology: 74.7% Total Sum of SVaMA SC Admission: 48.2% Admission SPMSQ Score: 45.9% Age: 45.5% mBI Admission Threshold > 30: 25.9% mBI Admission Threshold > 45: 17.2% Gender: 15.9%
**Performance Metrics**	Positive Predictive Value: 73.7% Sensitivity: 71.2% Negative Predictive Value: 69.1% Specificity: 71.7% Overall Accuracy: 86.4%

## Data Availability

The datasets used and/or analyzed during the current study will be made available upon reasonable request to the corresponding author (G.S.).

## Abbreviations

AI	Artificial intelligence
ANN	Artificial neural network
ADL	Activity of daily living
BI	Barthel Index
mBI	Modified Barthel Index
CND	Chronic neurological disease
ICF	International Classification of Functioning, Disability and Health
MLP	Multilayer perceptron
PCND	Patient with chronic neurological disease
PAOM	Physiatrist-assessed outcome measures
SD	Standard deviation
SPMSQ	Short portable mental status questionnaire
SVaMAsc	Sensory and Communication of the second part of the Multidimensional Assessment Schedule
